# Mesenchymal stem cells cultured in serum-free medium ameliorate experimental peritoneal fibrosis

**DOI:** 10.1186/s13287-021-02273-1

**Published:** 2021-03-23

**Authors:** Kohei Nagasaki, Ayumu Nakashima, Ryo Tamura, Naoki Ishiuchi, Kiyomasa Honda, Toshinori Ueno, Shigehiro Doi, Yukio Kato, Takao Masaki

**Affiliations:** 1 Department of Nephrology, Hiroshima University Hospital, 1-2-3 Kasumi, Minami-ku, Hiroshima, Hiroshima 734-8551 Japan; 2 Department of Stem Cell Biology and Medicine, Graduate School of Biomedical & Health Sciences, Hiroshima University, 1-2-3 Kasumi, Minami-ku, Hiroshima, Hiroshima 734-8553 Japan; 3 TWOCELLS Company, Limited, 16-35 Hijiyama-honmachi, Minami-ku, Hiroshima, 732-0816 Japan

**Keywords:** Mesenchymal stem cells, Serum-free culture condition, Immunosuppression, Peritoneal fibrosis, Peritoneal dialysis

## Abstract

**Background:**

Mesenchymal stem cells (MSCs) provide potential treatments for peritoneal fibrosis. However, MSCs cultured in media containing serum bring risks of infection and other problems. In this study, we compared the effect of human MSCs in serum-free medium (SF-MSCs) on peritoneal fibrosis with that of MSCs cultured in medium containing 10% fetal bovine serum (10%MSCs).

**Methods:**

Peritoneal fibrosis was induced by intraperitoneally injecting 0.1% chlorhexidine gluconate (CG). SF-MSCs or 10%MSCs were intraperitoneally administered 30 min after the CG injection. Ten days after the CG and MSC injections, we performed histological analyses and peritoneal equilibrium testing. In the in vitro experiments, we used transforming growth factor (TGF)-β1-stimulated human peritoneal mesothelial cells incubated in conditioned medium from MSCs to examine whether the SF-MSCs showed enhanced ability to produce antifibrotic humoral factors.

**Results:**

Histological staining showed that the SF-MSCs significantly suppressed CG-induced cell accumulation and thickening compared with that of the 10%MSCs. Additionally, the SF-MSCs significantly inhibited mesenchymal cell expression, extracellular matrix protein deposition and inflammatory cell infiltration. Peritoneal equilibration testing showed that compared with administering 10%MSCs, administering SF-MSCs significantly reduced the functional impairments of the peritoneal membrane. The in vitro experiments showed that although the conditioned medium from MSCs suppressed TGF-β1 signaling, the suppression did not significantly differ between the SF-MSCs and 10%MSCs.

**Conclusions:**

Serum-free culture conditions can enhance the antifibrotic abilities of MSCs by suppressing inflammation. Administering ex vivo expanded SF-MSCs may be a potential therapy for preventing peritoneal fibrotic progression.

## Background

By the end of 2016, 3.37 million patients worldwide had end-stage renal disease (ESRD) requiring renal replacement therapies such as hemodialysis (HD; 2.65 million patients), peritoneal dialysis (PD; 340,000 patients), and kidney transplants (740,000 patients). The number of ESRD patients is estimated to reach 5.44 million in 2030 [1]. Despite the annual cost for PD being lower than that for HD, recent studies have shown that patients who begin treatment with PD have a similar or lower risk of death than do patients who begin with HD [2, 3]. However, long-term PD causes peritoneal fibrosis due to bioincompatible dialysate components, uremic toxins, refractory or recurrent infectious peritonitis, and chronic inflammation [4]. Peritoneal fibrosis has no established preventive measures and remains a serious complication of long-term PD, leading to peritoneal dysfunctions such as peritoneal hyperpermeability and ultrafiltration failure [5]. The histological features of peritoneal fibrosis are a loss of peritoneal mesothelial cells, abnormal growth of α-smooth muscle actin (α-SMA)-positive myofibroblasts, marked collagen accumulation, and progressively increased submesothelial compact zone thickness [6]. Many studies have reported that transforming growth factor (TGF)-β1 has a pivotal role in the development of various fibrosis [7]. Our previous study showed that mesenchymal stem cells (MSCs) inhibit transforming growth factor (TGF)-β1 signaling in a paracrine manner and could be a potential treatment for peritoneal fibrosis [8].

MSCs are undifferentiated multipotent adult stem cells that are mainly collected from the bone marrow, adipose tissue, umbilical cord blood, and placenta and have broad anti-inflammatory and immunomodulatory properties [9–11]. MSCs have been studied extensively for over 30 years because of their interesting cell biology and broad clinical potential. Over 950 registered MSC clinical trials have been listed with the Food and Drugs Administration [12].

For clinical use of MSCs, ex vivo culturing is required to obtain sufficient numbers of optimally conditioned cells. Many previous studies cultured MSCs in media supplemented with fetal bovine serum (FBS) or human serum. Serum supplementation induces the risk of transmitting unknown viral diseases and provoking immune reactions [13, 14]. For example, animal-derived antigens and infectious agents in animal-derived growth supplements can be transmitted to patients undergoing MSC therapy [15, 16]. Furthermore, allogeneic human serum can arrest human MSC proliferation [17]. Therefore, animal-product-free and serum-free medium formulations are critical for clinical applications [18–20].

Human platelet lysate (hPL) has recently been used in place of animal products [10]. However, hPL can cause cell aggregation and infection and is difficult to ethically commercialize because of its human origin [21]. Our recent study suggested that STK2 (Kanto Reagents, Tokyo, Japan), a serum-free medium for MSCs, enhances the immunosuppressive and antifibrotic abilities of MSCs compared with that of hPL-containing media [22].

We recently showed that MSCs cultured in serum-free medium strongly suppressed inflammatory cell infiltration via enhanced expression of tumor necrosis factor-α-induced protein 6 (TSG-6) compared with MSCs cultured in medium containing 10% FBS in experimental renal fibrosis [22]. However, no studies have examined the effect of MSCs cultured in serum-free medium on peritoneal fibrosis; thus, we investigated the effect of MSCs cultured in serum-free medium on experimental peritoneal fibrosis.

## Methods

### Animals

Male 8-week-old Sprague-Dawley (SD) rats were purchased form Charles River Laboratories Japan (Yokohama, Japan). The rats were housed in a light- and temperature-controlled room in the Laboratory Animal Center of Hiroshima University (Hiroshima, Japan) with free access to food and water. The Animal Care and Use Committee at Hiroshima University approved all experimental protocols (permit number: A19–127), which were performed in accordance with the National Institutes of Health Guidelines on the Use of Laboratory Animals.

### MSCs and human peritoneal mesothelial cells (HPMCs)

Human MSCs derived from the bone marrow were obtained from Riken BRC (Ibaraki, Japan). MSCs at passage 4 were used in all experiments. MSCs cultured in STK2 and Dulbecco’s modified Eagle’s medium (DMEM; Sigma-Aldrich, St. Louis, MO, USA) containing 10% FBS (Sigma-Aldrich) were designated as “SF-MSCs” and “10%MSCs,” respectively. HPMCs were isolated from human omentum as previously described [23] and cultured in M199 medium (Life Technologies, New York City, NY, USA), including 10% FBS and penicillin/streptomycin (Nacalai Tesque, Inc., Kyoto, Japan). The Medical Ethics Committee of Hiroshima Graduate School of Biomedical Science permitted harvesting the omentum (E-84). Each patient provided written informed consent.

### Induction of peritoneal fibrosis and MSC treatment

After a 2-week acclimation, the SD rats were injected intraperitoneally with 3 mL of 0.1% chlorhexidine gluconate (CG) in 15% ethanol dissolved in saline. Thirty minutes after the CG injection, the rats were injected intraperitoneally with MSCs (5.0 × 10^6^ cells) suspended in 1 mL of phosphate-buffered saline.

### Peritoneal equilibrium test

Ten days after the CG and MSC injections, we performed peritoneal equilibrium testing before killing the rats. The rats were instilled intraperitoneally with PD solution (4.25% Dianeal; Baxter HealthCare, Deerfield, IL, USA) at 100 mL/kg body weight. After 30 min, peritoneal fluid and blood samples were collected via laparotomy and cardiac puncture, respectively, and the glucose and urea nitrogen concentrations were measured. The dialysate-to-plasma concentration ratio (D/P) of urea nitrogen (UN) and the dialysate-to-baseline dialysate concentration ratio (D/D0) of glucose represent the peritoneal permeabilities of blood urea nitrogen (BUN) and glucose.

### Histology and immunohistochemistry

Histological and immunohistochemical staining of paraffin-embedded 4-μm-thick tissue sections were performed as described previously [8]. The following primary antibodies were used: mouse monoclonal anti-α-SMA antibody (A2547; Sigma-Aldrich), rabbit polyclonal anti-TGF-β1 antibody (SAB4502954; Santa Cruz Biotechnology, Santa Cruz, CA, USA), rabbit polyclonal anti-collagen I antibody (ab34710; Abcam, Cambridge, UK), rabbit polyclonal anti-collagen III antibody (ab7778; Abcam), rabbit polyclonal anti-CD3 antibody (ab5690; Abcam), rabbit polyclonal anti-CD68 antibody (ab125212; Abcam), and rabbit polyclonal anti-CD163 antibody (ab182422; Abcam). Images of the microscopic sections were captured and measured using NIS-Elements (Nikon Corporation, Tokyo, Japan; × 200). The areas containing α-SMA, TGF-β1, collagen I, and collagen III were assessed in predetermined fields of the submesothelial compact zone, and the stained area was determined using Lumina Vision (Mitani, Osaka, Japan) in 50 fields from 5 rats.

### Cell cultures and treatments

To prepare the conditioned medium (CM) from both the 10%MSCs and SF-MSCs, cells were seeded into 10-cm dishes. When the cells reached 60–80% confluence, the medium was substituted with DMEM containing 0.1% FBS, followed by 48 h incubation. Then, each medium sample was collected and used as “CM from 10%MSCs” or “CM from SF-MSCs”. HPMCs were seeded into six-well plates and grown to subconfluence in M199 medium containing 10% FBS. The medium was then replaced with DMEM containing 0.1% FBS, CM from the 10%MSCs or CM from the SF-MSCs. After 12 h, HPMCs were treated with 2.5 ng/mL TGF-β1 (R&D Systems, Minneapolis, MN, USA) for 30 min or 24 h. Whole-cell lysates were prepared and subjected to western blot analysis.

### Water-soluble tetrazolium salts (WST)-1

MSCs (2.5 × 10^3^ cells/100 μL) were seeded into 96-well microplates and cultured in DMEM containing 10% FBS or STK2. After incubating for 0, 12, 24, and 48 h, 10 μL of WST-1 reagent (Takara Bio, Shiga, Japan) was added to each well and then incubated for 4 h. The absorbance was determined using a microplate reader at a test wavelength of 450 nm and a reference wavelength of 620 nm.

### Western blot analysis

Sample collection and western blotting were performed as previously described [8]. The following primary antibodies were used: rabbit polyclonal anti-phosphorylated Smad2 antibody (#3108; Cell Signaling Technology, Danvers, MA, USA), mouse monoclonal anti-Smad2 antibody (#3103; Cell Signaling Technology), anti-phosphorylated Smad3 antibody (#9520; Cell Signaling Technology), anti-Smad3 antibody (#9523; Cell Signaling Technology), mouse monoclonal anti-α-SMA antibody (A2547; Sigma-Aldrich), and mouse monoclonal anti-α-tubulin antibody (T9026; Sigma). The intensity of each band was quantified using ImageJ software (version 1.48p; National Institutes of Health, Bethesda, MD, USA).

### Quantitative real-time reverse-transcription PCR

RNA extraction and real-time reverse-transcription PCR were performed in accordance with previously described methods [22]. Specific oligonucleotide primers and probes for human TSG-6 (assay ID: Hs00200180_m1) and β-actin (assay ID: Hs01060665_g1) were obtained as TaqMan Gene Expression Assays (Applied Biosystems, Foster City, CA, USA). The mRNA levels were standardized by the level of β-actin.

### Statistical analysis

Statistical analysis was performed using the Mann–Whitney *U* test and Kruskal–Wallis test. *P* < 0.05 was considered statistically significant.

## Results

### MSCs cultured in serum-free medium suppressed peritoneal cell density and CG-induced thickening

Our previous study revealed that injecting 0.1% CG induced thickening of the submesothelial compact zone and that intraperitoneally administering MSCs cultured in DMEM containing 10% FBS (10%MSCs) suppressed this change [8]. Therefore, we examined whether SF-MSCs are more effective than 10%MSCs in an experimental peritoneal fibrosis model.

**Fig. 1:**
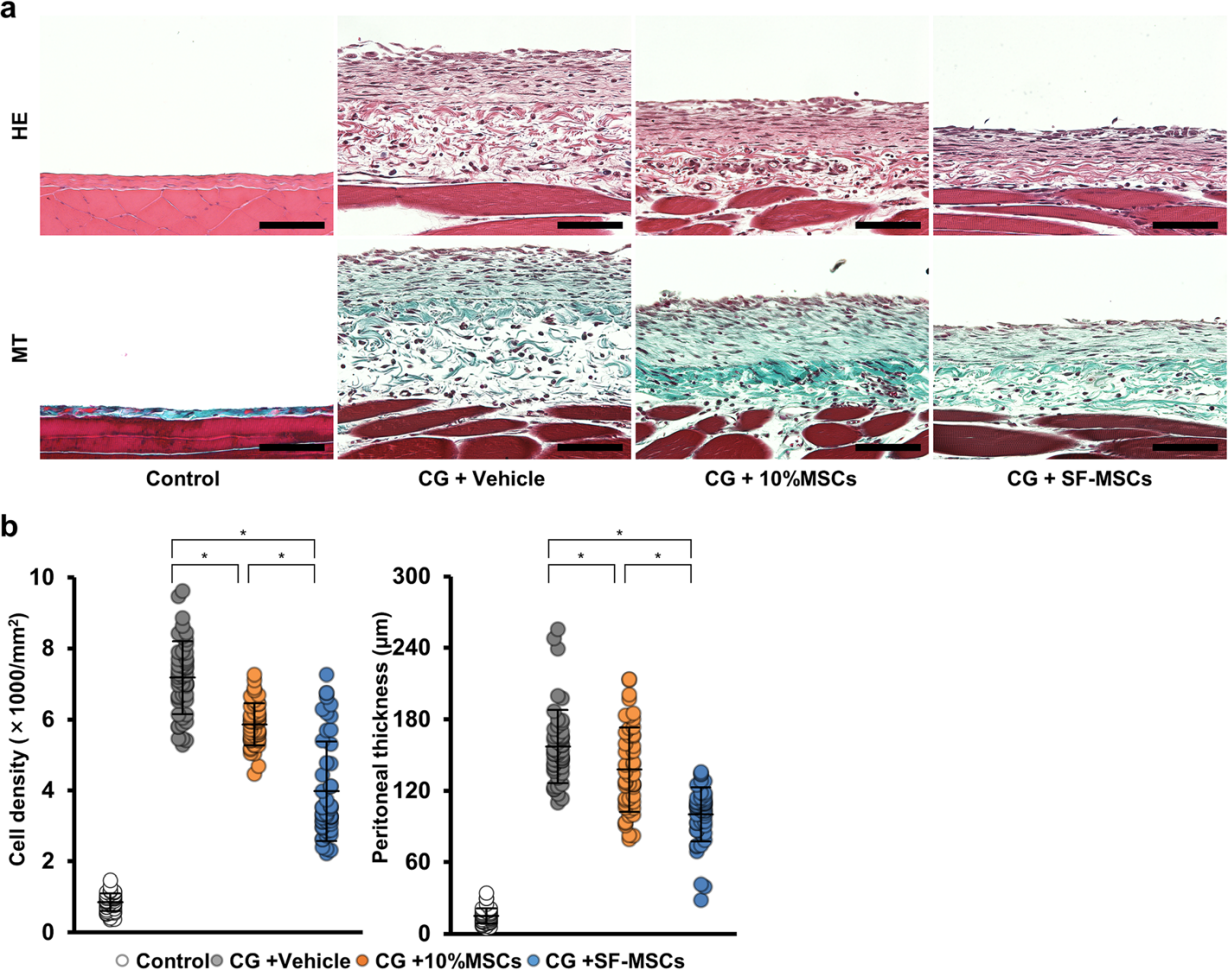
Effects of mesenchymal stem cell (MSC) injection on chlorhexidine gluconate (CG)-induced peritoneal cell density and thickness. Thirty minutes after injecting 0.1% CG, rats were intraperitoneally injected with MSCs cultured in DMEM containing 10% FBS (10%MSCs; 5 × 10^6^ cells) or MSCs cultured in serum-free medium (SF-MSCs; 5 × 10^6^ cells). After 10 days, the parietal peritoneum was examined via (**a**) hematoxylin-eosin staining and Masson’s trichrome staining to assess the peritoneal cell density and thickness, respectively. **b** Graph showing the cell density or thickness in each group. Control, control rats without CG injection; CG + vehicle, CG-injected rats treated with the vehicle; CG + 10%MSCs, CG-injected rats treated with 10%MSCs; CG + SF-MSCs, CG-injected rats treated with SF-MSCs. We examined 50 randomly chosen fields from 5 rats per group as described in the “Materials and Methods” section. *P < 0.05 (Kruskal–Wallis test)

Rats were injected with 0.1% CG in 15% ethanol dissolved in saline or 15% ethanol in saline as a control, then injected intraperitoneally 30 min later with either a vehicle or MSCs (5.0 × 10^6^ cells; *n* = 5 per rats group). On day 10, the rats were killed, and the parietal peritoneum was carefully dissected for histological examination. Histological changes in cell density and peritoneal thickness were assessed via hematoxylin-eosin and Masson’s trichrome staining, respectively. Both the cell density and thickness of the submesothelial compact zone were obviously increased microscopically in the CG-injected rats (vehicle group) compared with the non-injected rats (control group). These increases were significantly suppressed in the 10%MSC treatment group, and the suppression was even more significant in the SF-MSC treatment group (Fig. 1a, b).

### SF-MSCs suppressed fibrotic marker expression in rats with CG-induced peritoneal fibrosis

**Fig. 2:**
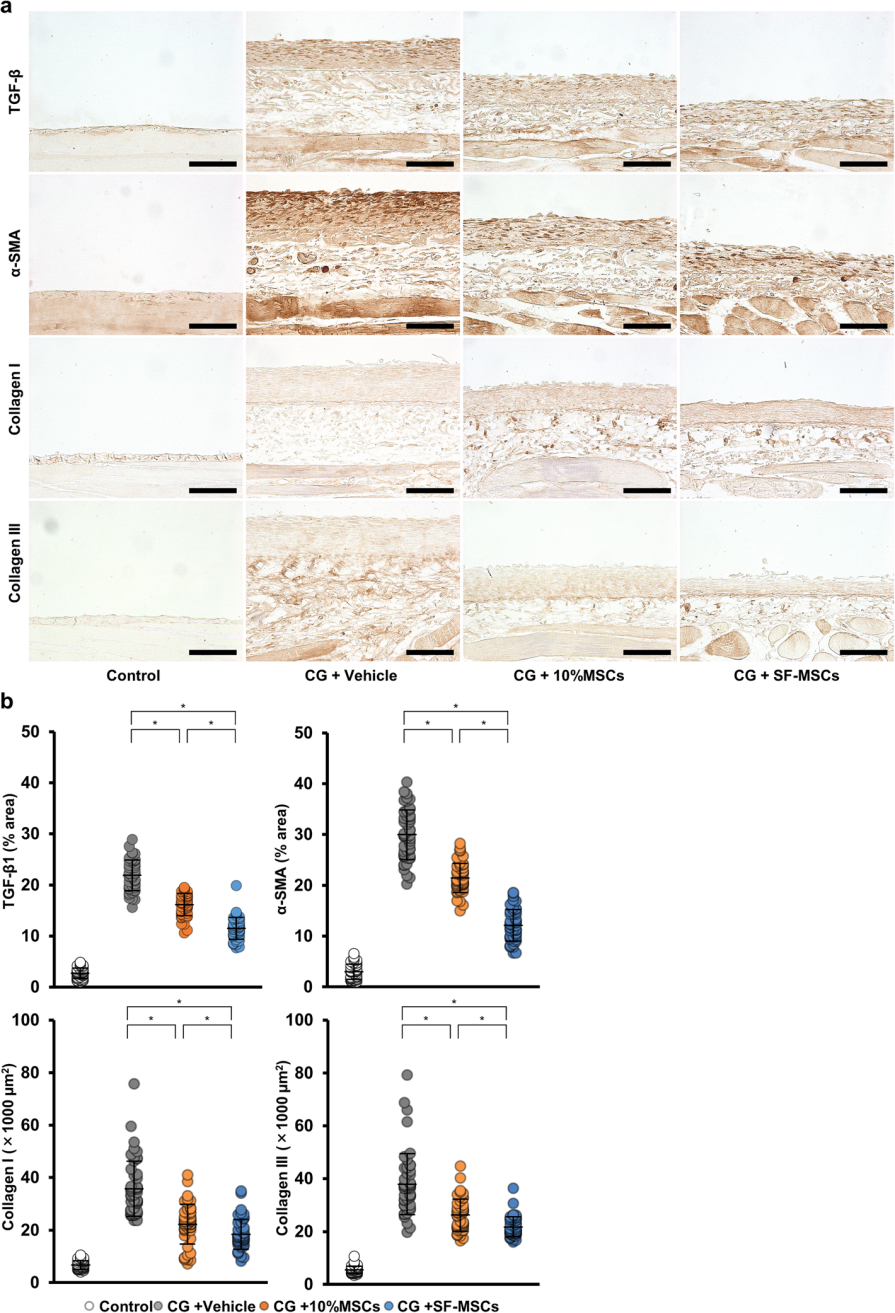
Effects of MSC injection on fibrosis markers and collagen expression in CG-induced peritoneal fibrosis. **a** Immunohistochemical analyses of TGF-β1, a profibrotic marker, α-smooth muscle actin (α-SMA), a marker for myofibroblasts, collagen I and collagen III expressions in peritoneal tissues were performed on day 10 after MSC injection. **b** Graph showing the percentage of TGF-β1-positive areas or α-SMA-positive areas, and the area of collagen-I-positive staining or collagen-III-positive staining, per group. We measure 50 fields from 5 rats per group. Abbreviations are as in Fig. 1. *P < 0.05 (Kruskal–Wallis test)

TGF-β1 is a profibrotic marker, and α-SMA is a marker of myofibroblasts. Therefore, we examined the peritoneal expressions of TGF-β1 and α-SMA via immunohistochemical analysis to assess CG-induced fibrosis. After the CG injection, TGF-β1 and α-SMA expressions were markedly increased in myofibroblasts in the upper layer of the submesothelial compact zone as well as in the vascular smooth muscle (Fig. 2a, b). MSC treatment, particularly SF-MSC treatment, significantly reduced the TGF-β1-positive and α-SMA-positive areas that had been increased by CG.

### SF-MSCs suppressed the extracellular matrix proteins in rats with CG-induced peritoneal fibrosis

Collagen types I and III, which are secreted by fibroblasts and other mesenchymal cells, are markers of extracellular matrix proteins and indicators of fibrosis. Specifically, collagen type III plays an important role in various inflammation-associated pathologies. Thus, we next investigated the expressions of collagen types I and III in the submesothelial compact zone. The collagen-I-positive and collagen-III-positive areas were obviously increased in the CG-injected rats compared with those in the control rats. These increases were significantly suppressed in the 10%MSC group, and the suppression was even more pronounced in the SF-MSC group (Fig. 2a, b). These findings together with the results in Fig. 1 indicate that MSC administration suppressed peritoneal fibrosis in CG-injected rats, and the SF-MSCs more strongly suppressed peritoneal fibrosis than did the 10%MSCs.

### SF-MSCs inhibited inflammatory cell infiltration in rats with CG-induced peritoneal fibrosis

**Fig. 3:**
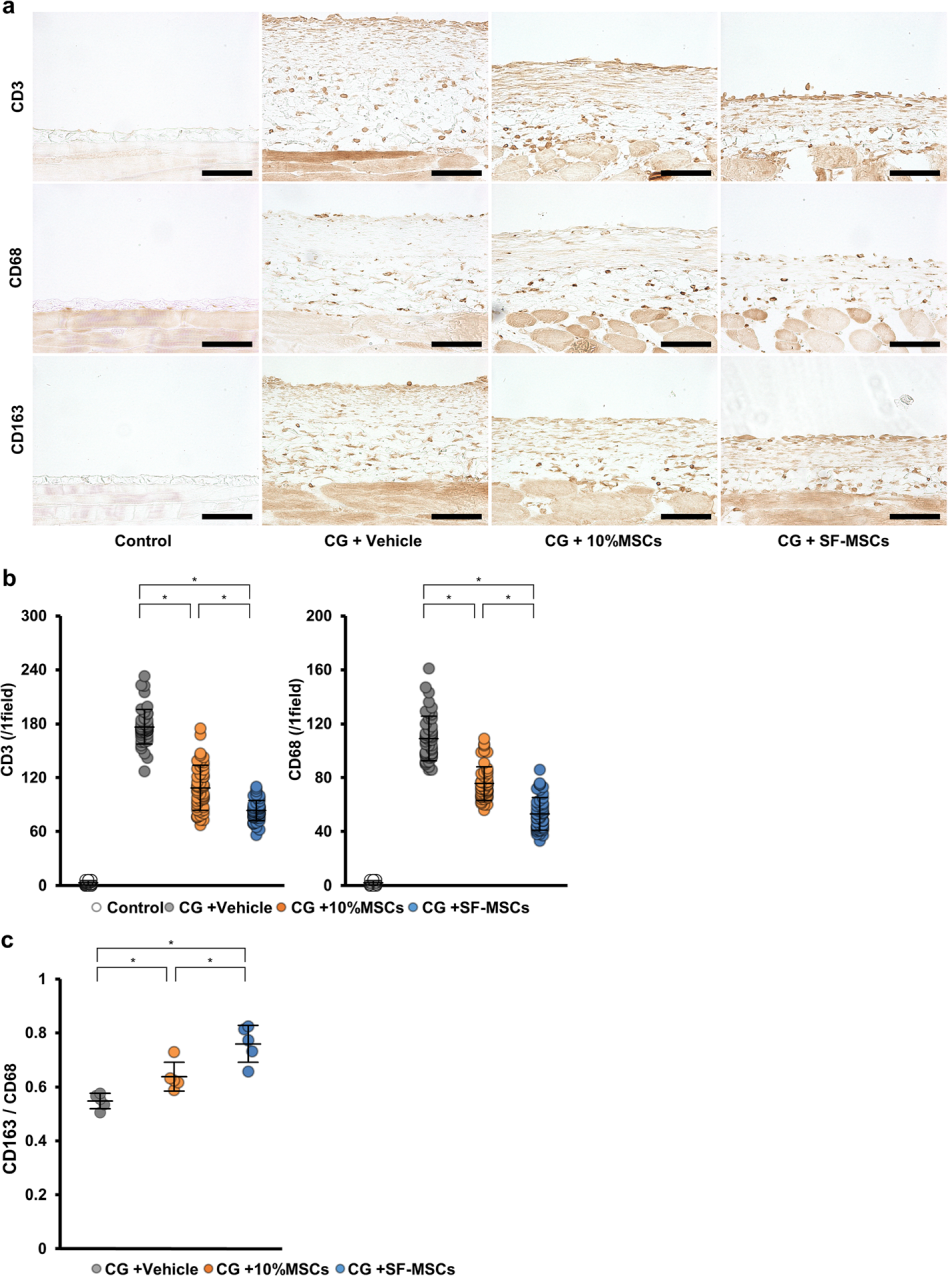
Effects of MSC injection on CD3+ T-lymphocyte and macrophage infiltration and on M2 macrophage polarization in CG-induced peritoneal fibrosis. **a** Immunohistochemical analyses for CD3+ T lymphocytes, CD68+ macrophages, and CD163+ M2 macrophages. We measured 50 fields from 5 rats per group. **b** Graph showing the number of CD3- and CD68-positive cells per group. **c** For assessing the polarization into CD163+ M2 macrophages, the ratio of CD163+ cells (M2 macrophages) to CD68+ cells (total macrophages) were calculated for each rat (*n* = 5 rats per group). Abbreviations are as in Fig. 1. *P < 0.05 (Kruskal–Wallis test)

Persistence of inflammatory cell infiltration, such as infiltration of T lymphocytes and macrophages, plays an important role in the fibrotic process. To examine inflammatory cell infiltration, we analyzed the peritoneal expressions of CD3 (a T-lymphocyte marker), CD68 (a macrophage marker), and CD163 (an M2 macrophage marker) via immunostaining. The numbers of CD3-positive and CD68-positive cells were increased in the peritoneums of CG-injected rats compared with those of the control rats. These increases were significantly suppressed in the 10%MSC-treated group and even more significantly suppressed in the SF-MSC-treated group (Fig. 3a, b).

Our previous study revealed that SF-MSCs significantly induced polarization from the proinflammatory M1 to the immunosuppressive M2 macrophage phenotype [22]. To further evaluate the polarization rate into M2 macrophages, we calculated the ratio of M2 macrophages (CD163-positive cells) to total macrophages (CD68-positive cells) (Fig. 3c). The number of M2 macrophages was significantly increased in the 10%MSC group, and the increase was more pronounced in the SF-MSC group. Thus, MSC treatment suppressed the inflammatory cell infiltration, and SF-MSC treatment more effectively suppressed the infiltration than did the 10%MSC treatment. Additionally, SF-MSCs strongly suppressed the persistence of inflammation via enhanced induction of a phenotype change from proinflammatory M1 to immunosuppressive M2 macrophages.

### SF-MSCs reduced the functional impairments of the peritoneal membrane in rats with peritoneal fibrosis

**Fig. 4:**
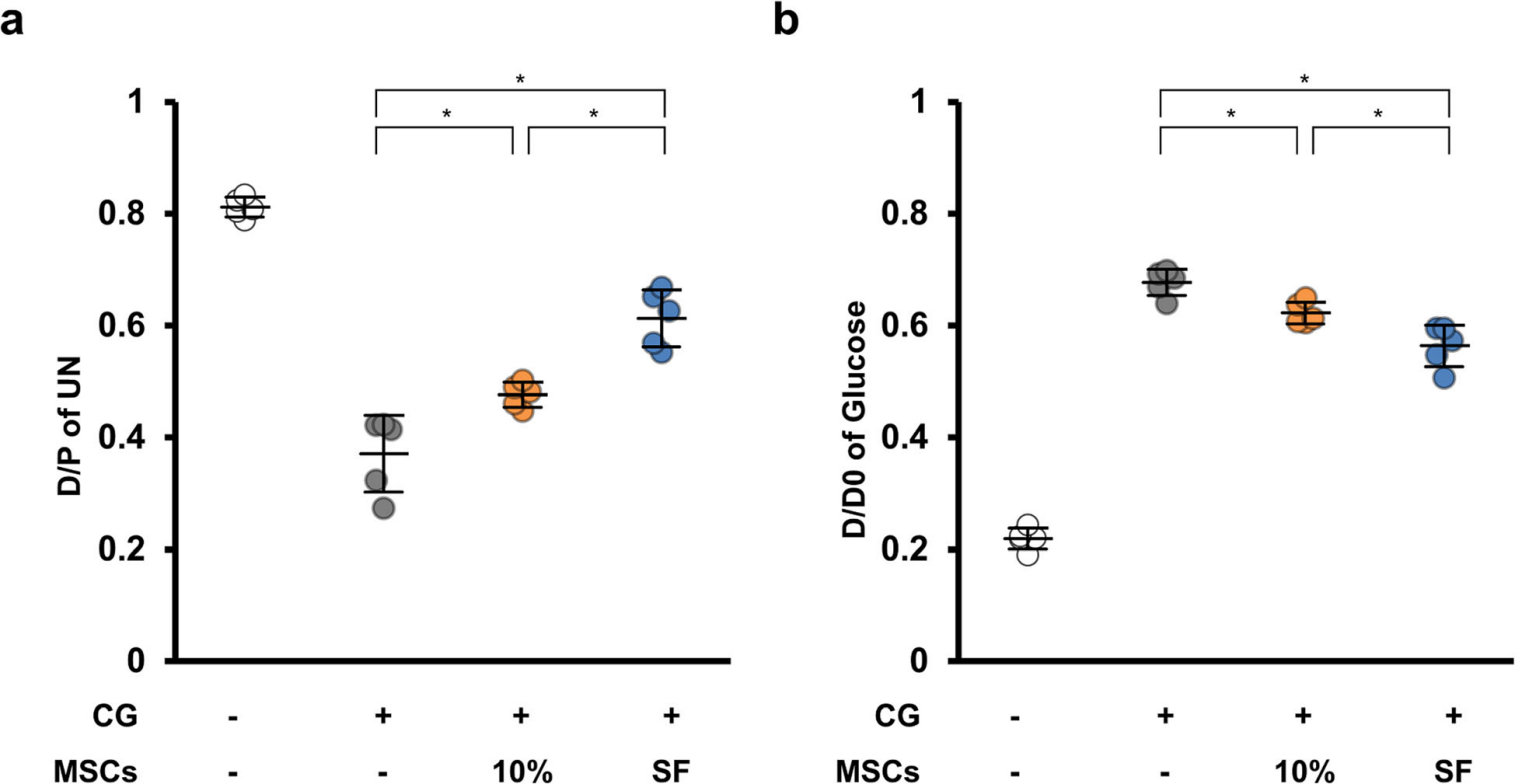
Effects of MSC injection on CG-induced peritoneal membrane dysfunction. Rats were instilled with PD solution (4.25% dialysis solution) at 100 mL/kg body weight. After 30 min, PD solution and plasma were collected. **a** Dialysate-to-plasma concentration ratio for urea nitrogen (D/P of UN); **b** dialysate-to-baseline dialysate concentration ratio for glucose (D/D0 of glucose). Abbreviations are as in Fig. 1. *n* = 5 rats per group. *P < 0.05 (Kruskal–Wallis test)

To assess the functional alteration of the peritoneal membrane, we performed peritoneal equilibrium testing. We calculated the ratio of the dialysate creatinine (or UN) to the plasma creatinine (D/P) and the ratio of glucose dialysate levels to the initial glucose dialysate levels (D/D0). If peritoneal dysfunction progresses, the D/P increases, and the D/D0 decreases owing to peritoneal hyperpermeability and ultrafiltration failure. CG injection increased the UN D/P, while MSC treatment, particularly SF-MSC treatment, significantly reduced it (Fig. 4). Conversely, CG injection reduced the D/D0 of glucose. Although MSC treatment did not significantly restore the reduced D/D0, the SF-MSC treatment significantly increased it compared with the 10%MSC treatment. Therefore, CG-injected rats treated with SF-MSCs presented improved BUN transport rates from the plasma and absorption rates of glucose from the dialysate. Thus, administering MSCs, especially SF-MSCs, can improve functional impairments in the peritoneal membrane.

### Conditioned medium from MSCs prevented fibrotic changes in HPMCs by inhibiting TGF-β1-induced phosphorylation of Smad2 in vitro

**Fig. 5:**
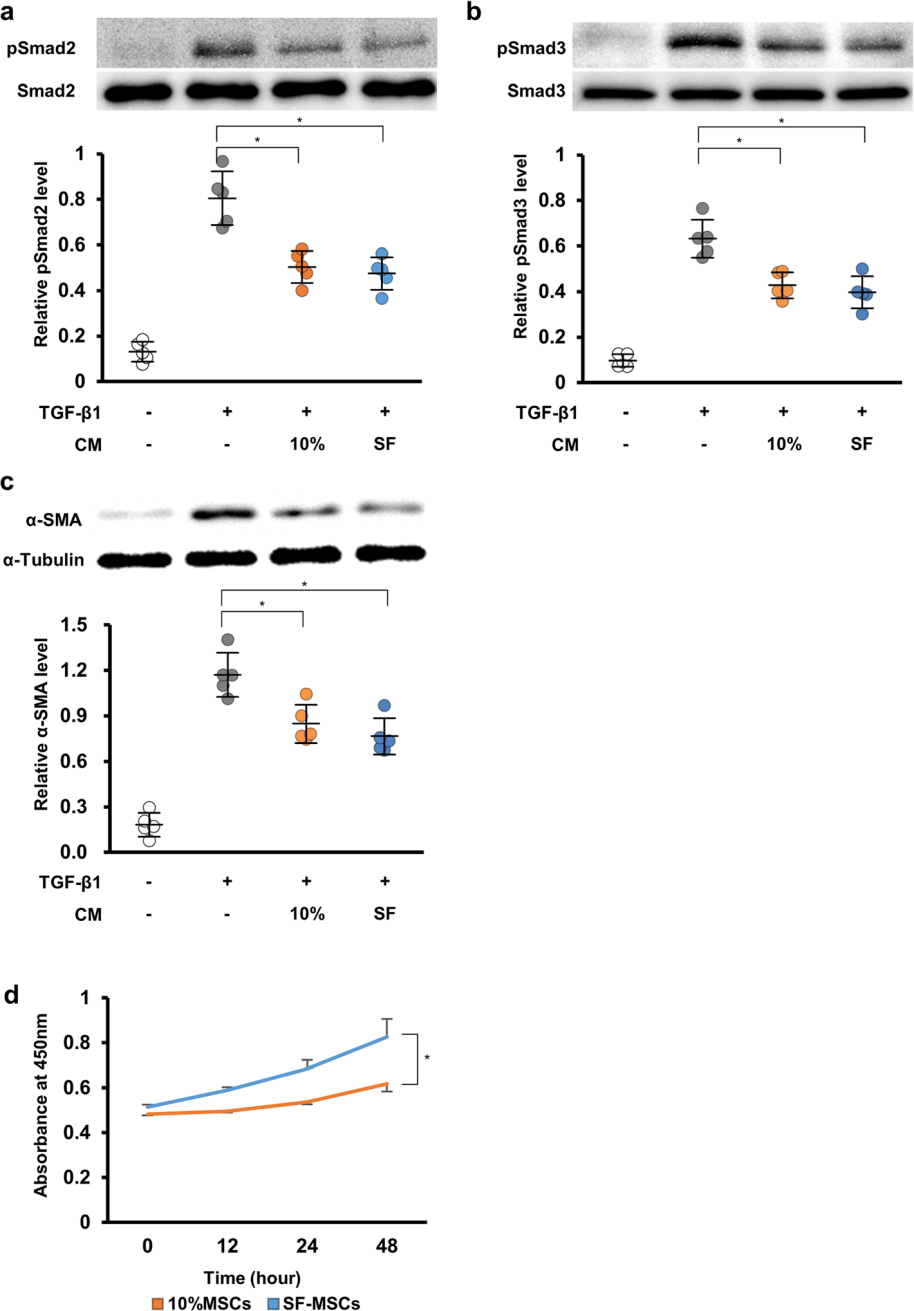
Effects of conditioned medium (CM) from MSCs on the TGF-β1/Smad signaling pathway and the proliferative ability of MSCs cultured in STK2 serum-free medium or 10% FBS-containing medium. HPMCs were grown, then the medium was replaced with CM from 10%MSCs, CM from SF-MSCs, or control medium. After 12 h, HPMCs were treated with TGF-β1 for 30 min (pSmad2, pSmad3) or 24 h (α-SMA), and whole-cell lysates were prepared and subjected to western blot analysis. Western blot analysis of **a** pSmad2, **b** pSmad3, and **c** α-SMA expression. Graph showing the densitometric analysis of pSmad2, pSmad3, and α-SMA expression normalized to Smad2, Smad3, and GAPDH expression. *n* = 5 per group. *P < 0.05 (Kruskal–Wallis test). **d** MSCs were cultured in DMEM containing 10% FBS or STK2 for 0, 12, 24, and 48 h. Surviving cells were subsequently assessed via WST-1 assay. Graph showing the absorbance value at each time point. *n* = 5 per group. *P < 0.05 (Mann–Whitney *U* test)

Next, we measured pSmad2 and α-SMA to examine whether SF-MSCs could more effectively suppress fibrosis in HPMCs than 10%MSCs could. We prepared CM from human MSCs cultured in STK2 serum-free medium or 10% FBS-containing medium. HPMCs were grown to subconfluence, then the medium was replaced with CM from 10%MSCs, CM from SF-MSCs, or control medium. After 12 h, HPMCs were treated with TGF-β1 for 30 min or 24 h, and whole-cell lysates were prepared and subjected to western blot analysis. The pSmad2 and pSmad3 levels were increased via TGF-β1 treatment. The CM from MSCs significantly suppressed these increases with no significant difference between CM from 10%MSCs and CM from SF-MSCs (Fig. 5a, b). Similar results were observed for α-SMA protein expression (Fig. 5c).

### STK2 serum-free medium enhanced the MSC proliferative activity

To investigate whether STK2 serum-free medium could enhance the proliferative ability of MSCs compared with 10% FBS-containing medium, we examined the proliferative activity of the SF-MSCs and 10%MSCs via WST-1 assay. Proliferative activity was confirmed by evaluating the absorbance value that showed surviving cells. The absorbance value of the SF-MSCs increased more significantly than did that of the 10%MSCs over time, indicating that STK2 serum-free medium enhanced the MSC proliferative ability (Fig. 5d).

### STK2 serum-free medium enhanced the expression of TSG-6 in MSCs

**Fig. 6:**
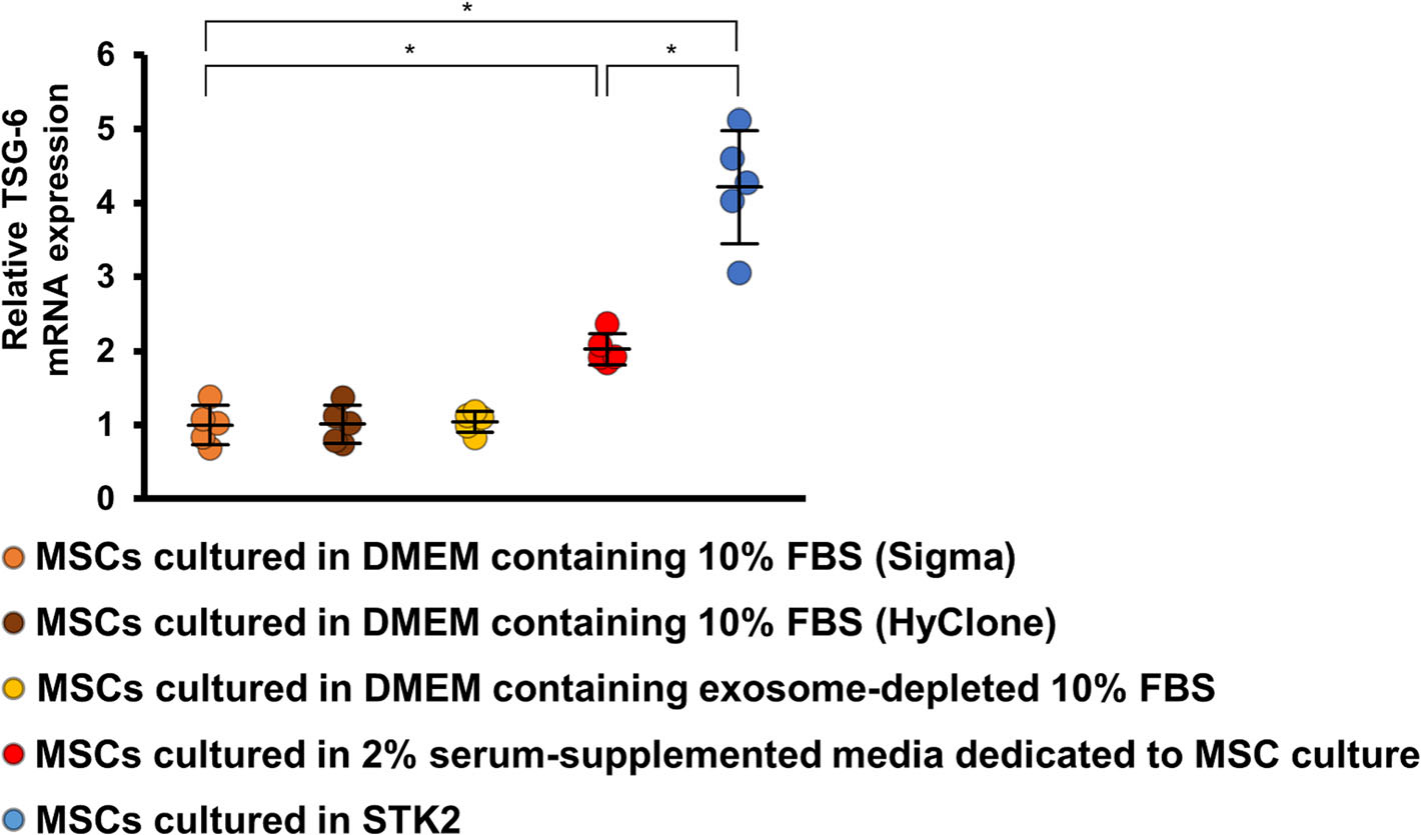
Effects of culture medium on TSG-6 expression in MSCs. MSCs were cultured in the following media: DMEM containing 10% FBS (Sigma), DMEM containing 10% FBS (HyClone), DMEM containing exosome-depleted 10% FBS (System Biosciences), 2% serum-supplemented media dedicated to MSC culture (Promo Cell), and STK2 serum free medium. Total RNA was extracted using TRIzol Reagent. Graph showing the TSG-6 mRNA level normalized to β-actin mRNA level. *n* = 5 per group. *P < 0.05 (Kruskal–Wallis test)

Several studies have reported that MSCs secrete TSG-6, which plays an important role in suppressing, at an early phase, the infiltration of inflammatory cells induced by tissue injury [22, 24]. We found that the TSG-6 mRNA level in MSCs cultured in 2% serum-supplemented media dedicated to MSC culture (Promo Cell, Heidelberg, Germany) increased compared with 10%MSCs, and further upregulation was observed in SF-MSCs (Fig. 6). Moreover, the TSG-6 mRNA level was similar between MSCs cultured in DMEM containing 10% FBS from two different manufacturers (Sigma and HyClone, Logan, UT, USA) and MSCs cultured in DMEM containing exosome-depleted 10% FBS (EXO-FBS; System Biosciences, Palo Alto, CA, USA).

## Discussion

Peritoneal fibrosis is histologically characterized by excess accumulation of extracellular matrix components, such as collagen, and proliferation of myofibroblasts in the peritoneal mesothelium [25]. In this study, MSC-induced suppression of peritoneal fibrosis was evidenced by a significant reduction in cell density and peritoneal thickening as well as suppression of collagen deposition and inflammatory cell accumulation. Although direct antifibrotic effects of the SF-MSCs were similar to those of the 10%MSCs, SF-MSC administration ameliorated the CG-induced inflammation and peritoneal fibrosis in the rats to a greater extent than did 10%MSC administration.

Infiltration of immune cells, such as macrophages and lymphocytes, contributes to development of peritoneal fibrosis. These infiltrated immune cells secrete profibrotic cytokines, such as TGF-β1, which is the main signal conductor for development of peritoneal fibrosis [26]. Additionally, bioincompatible dialysate components activate the TGF-β1-dependent signaling pathway [27]. TGF-β1 activates many downstream molecules in profibrotic signaling, including Smad signals, which promote peritoneal fibrosis [10, 28]. Intraperitoneal administration of CG caused the macrophages and lymphocytes to infiltrate the peritoneum, and MSC administration inhibited this infiltration. Mechanistically, MSCs reportedly exhibit high TSG-6 expression, which inhibits inflammatory cell migration by affecting chemokine receptors and attenuates inflammatory cascades in the early inflammatory phase at damaged sites [29, 30]. Our study showed that SF-MSCs significantly increased TSG-6 expression compared with not only 10%MSCs but also MSCs cultured in 2% serum-supplemented media dedicated to MSC culture. Additionally, compared with 10%MSCs, SF-MSCs more effectively induced conversion from the M1 to M2 phenotype in macrophages [22]. M1 macrophages exert proinflammatory effects by producing proinflammatory cytokines and proteolytic enzymes, whereas M2 macrophages have anti-inflammatory effects and contribute to inflammation resolution and tissue remodeling [31]. In addition to these beneficial effects, STK2 serum-free medium enhanced the proliferative activity of MSCs better than the 10% FBS-containing medium. This is because STK2 contains growth factors, including FGF2, insulin, PDGF, and EGF, and lipids, including fatty acids and phospholipids, in addition to nutrients and minerals [22]. In particular, FGF2 is presumed to be the key growth factor that contributes to the strong enhanced proliferation of MSCs [32].

SF-MSC administration more significantly reduced peritoneal TGF-β1 levels than did 10%MSC administration. Additionally, compared with 10%MSCs, SF-MSCs increased the M2/total macrophage ratio at damaged sites. However, in vitro experiments showed that although the CM from MSCs suppressed TGF-β1 signaling, this suppression did not significantly differ between SF-MSCs and 10%MSCs. Additionally, our previous studies showed that injected MSCs were detected on the peritoneal surface until day 3 in rats following CG-induced peritoneal fibrosis, and in experimental renal fibrosis, serum-free culturing did not promote MSC migration or engraftment [22]. Thus, the direct antifibrotic effect of SF-MSCs is similar to that of 10%MSCs, and the strong fibrosis-suppressing effect of SF-MSCs is due to suppression of inflammation.

Many long-term PD patients have progressive peritoneal fibrosis and angiogenesis, which causes increased solute transport and loss of ultrafiltration [5]. PD patients generally undergo positron emission tomography (PET) to characterize solute transport and ultrafiltration. PET results can help evaluate the risk of peritoneal dysfunction and optimize PD prescriptions [33]. Our PET demonstrated that SF-MSC administration suppressed peritoneal dysfunction, such as a low D/P of UN or a high D/D0 of glucose, more effectively than did 10%MSC administration. A low UN D/P demonstrates increased solute transport and a high D/D0 of glucose demonstrates loss of ultrafiltration. Therefore, MSC administration holds great promise for suppressing progression of clinical peritoneal dysfunction by ameliorating inflammation and peritoneal fibrosis, and SF-MSCs are more effective than are 10%MSCs.

In clinical applications, using serum-free media for culturing MSCs has many advantages including eliminating the need to check for differences in serum lots, shortening the culture period, more efficient and stable cell proliferation, and reducing the risk of infection from serum-derived components among others. Based on these considerations and the results from our study, we strongly believe that SF-MSCs are a promising treatment for suppressing peritoneal fibrosis. PD patients must exchange dialysate several times daily and are chronically exposed to causative dialysate components. Therefore, predicting when inflammation, such as peritonitis, will occur is difficult. Future studies should evaluate the MSC administration period, administration method, and dosage.

## Conclusion

In rat models of peritoneal sclerosis, MSCs cultured in serum-free medium can more effectively suppress fibrosis than can MSCs cultured in serum-containing medium. Thus, administering ex vivo expanded MSCs in serum-containing medium may be a useful therapy for preventing peritoneal dysfunction.

## Data Availability

The data that support the findings of this study are available from the corresponding author upon reasonable request.

## Abbreviations

10%MSCs: Mesenchymal stem cells cultured in medium containing 10% fetal bovine serum
CG: Chlorhexidine gluconate
CM: Conditioned medium
D/D: Dialysate-to-baseline dialysate concentration ratio
DMEM: Dulbecco’s modified Eagle’s medium
D/P: Dialysate-to-plasma concentration ratio
FBS: Fetal bovine serum
hPL: Human platelet lysate
HPMCs: Human peritoneal mesothelial cells
MSCs: Mesenchymal stem cells
PD: Peritoneal dialysis
SD: Sprague-Dawley
SF-MSCs: Mesenchymal stem cells cultured in serum-free medium
SMA: Smooth muscle actin
TGF: Transforming growth factor
TSG-6: Tumor necrosis factor-α-induced protein 6
UN: Urea nitrogen
WST: Water-soluble tetrazolium salts
